# Cancer as a Proinflammatory Environment: Metastasis and Cachexia

**DOI:** 10.1155/2015/791060

**Published:** 2015-10-05

**Authors:** Nelson Inácio Pinto, June Carnier, Lila M. Oyama, Jose Pinhata Otoch, Paulo Sergio Alcântara, Flavio Tokeshi, Claudia M. Nascimento

**Affiliations:** ^1^Departamento de Fisiologia, Universidade Federal de São Paulo, 04023-062 São Paulo, SP, Brazil; ^2^Universidade de São Paulo, Clínica Cirúrgica, Hospital Universitário, 05508-000 São Paulo, SP, Brazil

## Abstract

The development of the syndrome of cancer cachexia and that of metastasis are related with a poor prognostic for cancer patients. They are considered multifactorial processes associated with a proinflammatory environment, to which tumour microenvironment and other tissues from the tumour bearing individuals contribute. The aim of the present review is to address the role of ghrelin, myostatin, leptin, HIF, IL-6, TNF-*α*, and ANGPTL-4 in the regulation of energy balance, tumour development, and tumoural cell invasion. Hypoxia induced factor plays a prominent role in tumour macro- and microenvironment, by modulating the release of proinflammatory cytokines.

## 1. Introduction

The malignancy grade of a tumour is fundamentally associated with both the development of metastatic lesions, a complex process that involves the propagation of cancer cells from the primary site to distant organs, and cancer-associated cachexia, which is dependent on factors produced by either the tumour microenvironment (tumour cells and surrounding tissues) or by the macroenvironment (comprising tumour released substances in the circulation and the secretion of proinflammatory factors by other tissues).

Cancer cachexia is an inflammatory condition, as is obesity. An excess of adipose tissue depots is associated with an increased risk of the incidence of some types of cancer and increased morbidity and mortality in cancer patients. Some factors such as insulin-like growth factors, leptin, and steroid hormones seem to connect obesity and cancer, once they contribute to the development of the chronic inflammatory state [1]. The increased estrogen synthesis associated with obesity in postmenopausal women augments the risk of development of postmenopausal breast and endometrial cancer. The constant presence of a high concentration of insulin in blood related to body fat excess and the increase of bioactive fractions of insulin, such as growth factor 1, via the downregulation of levels of insulin like growth factor-binding proteins 1 and 2, contribute to increased risk of cancer development [2, 3]. Adipose stromal cells in WAT may present a role in cancer development and progression, promoting changes in the production of adipokines. Proinflammatory cytokines are involved in cancer-inducing mechanisms, while adiponectin could have anticancer effects [3, 4]. The inflammatory tumour microenvironment is amplified by infiltrating macrophages, further increasing the production of cytokines, prostaglandins, angiogenic factors, and metastasis [4].

The details of the multi-step process of metastasis have been described by others [5, 6]. Here, we will provide only a brief synopsis. In the primary tumour microenvironment, epithelial cancer cells are surrounded by pericytes, various extracellular matrix (ECM) proteins, and cancer-associated fibroblasts (CAF), which include fibroblasts, macrophages, endothelial cells, lymphocytes, and granulocytes. This tumour microenvironment produces various factors, such as TNF*α*, TGF*β*, Wnt, and HIF-1*α*, all of which stimulate a transient epithelial-mesenchymal transition (EMT) to promote cancer progression, invasion, and metastasis. EMT is a phenomenon wherein cells loose epithelial properties and gain mesenchymal properties, causing the loss of cell-cell contact and cell polarity. EMT increases cell migratory and invasive abilities, which are associated with the first step of cancer metastasis.

Cytokines such as TNF*α* and interleukins, which are produced by both tumour cells and surrounding cells trigger NF-*κ*B signalling [4], consequently causing the release of TGF-*β*, an inducer of EMT [7, 8].

For the last decade, white adipose tissue has been considered an important endocrine organ producing several humoral factors including leptin, adiponectin, TNF-*α*, IL-6, zinc-a2 glycoprotein (ZAG), IL-10, plasminogen activator inhibitor-1, and visfatin. These adipokines participate in the regulation of energy balance and a several physiological processes, including insulin sensitivity and the inflammatory response [9].

In fact, cachexia is frequently accompanied by food intake reduction and increases in proinflammatory factors such as C-reactive protein, TNF-*α*, IL-6, and IL-1 [10].

Several reports have shown [10] that white adipose tissue (WAT) expresses and secretes proinflammatory factors in a rodent model of cancer cachexia, suggesting that these adipokines could be involved in the systemic inflammation in cancer patients.

In this review, we discuss some factors from the micro- and macroenvironment that could contribute to the development of metastasis and cancer cachexia.

## 2. Cancer Cachexia

Metabolic disorders that develop in anorexia and cancer cachexia are different. The weight loss in anorexia occurs mainly by the weight loss from fat, whereas cachexia involves significant weight loss from fat and muscle mass [50]. Anorectic patients respond to nutritional treatment more effectively than cachexia. In opposition, in the process of cancer cachexia, muscle mass cannot be maintained and food intake frequently cannot reverse this condition [51, 52].

Cancer cachexia is a multifactorial syndrome characterized by a persistent and involuntary loss of skeletal muscle mass and fat mass, which contributes to progressive functional damage. This condition is related to a negative protein and energy balance, caused by a variable combination of abnormal metabolism and reduced food intake; conventional nutritional support is not enough to improve it. In addition, increased lipolysis and insulin resistance and reduced physical activity are present [52].

Cachexia may have a high prevalence in patients with cancer, depending on the stage of the disease and the type of tumour. The range of cachexia in patients with advanced cancer is from 60 to 80%. Pancreatic and gastric cancers are among the cancers with the highest prevalence of cachexia, approximately 85%. Over 20% of cancer patients die from cachexia [53].

The cancer in digestive tract frequently causes undernutrition related to the obstruction of alimentary tract, constipation, maldigestion, and malabsorption. However, the cachexia present in these patients was not only associated with malnutrition, since in these patients the metabolism differs from patients with anorexia, as the former present high proteolysis associated with an increase in proinflammatory protein and cytokines, aspects that will be discussed below [54].

Cancer cachexia progression comprises a variety of phases, including precachexia, cachexia, and refractory cachexia. Refractory cachexia is more difficult to reverse and is characterized by active catabolism or the presence of factors produced by the tumour and other tissues that do not allow an improvement in weight loss. This stage of cachexia is observed in patients who have a rapid progression of cancer that conventional cancer therapy cannot affect or in patients in the terminal stages of cancer, as in the cachexia consensus published in 2011 [52].

The type and stage of cancer, cancer treatment, food intake, and systemic inflammation are factors that can influence the progression from precachexia to cachexia [52, 55].

Precachexia is considered the initial stage in which clinical and metabolic abnormalities, such as glucose intolerance and anorexia, already occur and precede an involuntary weight loss (≤5%). Cachexia is identified in patients with equivalent or more than 5% of body weight loss during the previous 6 months, or a BMI below 20 kg/m² and a continuous weight loss of more than 2%, or continuous weight loss (<2%) and sarcopenia. For many years, the majority of researches focus on muscle proteolysis in cancer cachexia. Nowadays, both muscle and adipose tissue wasting are considered important for development of cachexia in cancer patients [52].

Several factors produced during cancer development either by the micro- or the macroenviroment promote a proinflammatory condition, which are related to alterations on the regulation of energy balance. We will discuss some factors that are potentially involved in the development of cancer cachexia and metastasis.

## 3. Ghrelin

Energy balance results from the amount of food intake and the total energy expenditure. The control of energy balance depends on a complex network system regulated by several peripheral and central signals [56]. Cancer patients with systemic inflammation show a decrease in food intake, and hypermetabolism has also been described [52].

Ghrelin is a hormone produced mainly by enteroendocrine cells and, to a lesser extent, in the colon, hypothalamus, pituitary, endocrine pancreas, placenta, lung, cardiomyocytes, ovaries, and testes. Additionally, several studies have demonstrated that ghrelin mRNA and protein are expressed in many cancer and tumour tissues (see review Chopin et al., 2012) [11]. Ghrelin was first reported as an important stimulator of hunger and an inhibitor of energy expenditure [57]. Many studies have been conducted to verify the relationship between the decreased appetite present in cancer patients and the hormones that stimulate hunger, such as ghrelin.

These studies found high levels of total ghrelin and acylated ghrelin in most cachectic cancer patients compared with cancer patients without cachexia and patients without cancer. These results were demonstrated in patients with several types of cancer, such as breast, colon, and lung cancers [11].

However, interestingly, cancer cachexia patients did not show increased appetite, despite their increased ghrelin levels. It seems that, in these patients, ghrelin levels increased in an attempt to reverse the catabolic state, as a compensatory mechanism [11, 58].

Chopin et al. (2012) [11] suggested that ghrelin resistance is similar to the GH-resistant state observed in cancer cachexia patients because GH levels are elevated, whereas IGF-I levels are not.

In contrast, Fujitsuka et al. (2011) [59] evaluated mice inoculated with AH-130 ascites hepatoma cells, which cause cachexia, and found lower plasma concentrations of ghrelin and reductions in the expression of hypothalamic Y (NPY) neuropeptide, agouti-related peptide, and proopiomelanocortin (POMC) compared with animals without cancer cachexia. It appears that the reduction in ghrelin occurs due to excessive hypothalamic interactions between serotonin (5-HT) and corticotropin-releasing factor, which are stimulated by proinflammatory cytokines. In addition, tumour-bearing rats showed attenuated responses of increasing food intake when ghrelin was administered intravenously, indicating possible ghrelin resistance. This finding could partially explain why cancer cachexia patients present hypophagia despite their low levels of leptin and high levels of ghrelin.

In addition to investigations of the effects of ghrelin on energy balance, this hormone was evaluated to determine whether it could have a role in tumour growth, because ghrelin stimulates growth hormone secretion. Accordingly, Northrup et al. (2013) [60] showed that both ghrelin and anamorelin, an active agonist of the ghrelin receptor, did not cause an increase in tumours in tumour-bearing nude mice. Despite this result, the influence of ghrelin on tumour growth remains to be elucidated. As stated by Chopin et al. (2012) [11], it is controversial whether ghrelin has tumour-promoting effects or could inhibit tumourigenesis* in vivo*. Further studies are needed to better understand the effects of ghrelin on cancer cachexia development.

The administration of ghrelin as a treatment for cancer cachexia patients has been evaluated [59].* In vitro* evidence showed that the expression of atrogenes through PI3K*β*-, mTORC2-, and p38-mediated pathways in myotubes and dexamethasone-induced muscle atrophy was inhibited by acylated and nonacylated ghrelin, suggesting that this peptide may be able to prevent muscular atrophy [12]. Additionally, ghrelin inhibits myostatin secretion, a negative regulator of skeletal muscle mass [61].

## 4. Myostatin

Myostatin expression is upregulated in experimental models of cancer cachexia [13, 14]. In humans, blood and muscle myostatin levels are upregulated in gastric cancer patients [62]. Myostatin, also known as growth/differentiation factor-8 (GDF-8), was described in 1997 and has been shown to be a potent negative regulator of muscle growth. This protein is expressed in muscle and other tissues, such as heart, adipose tissue, and mammary gland [63].

Active myostatin binds the activin type II B receptor (ActRIIB) and, to a lesser extent, the related ActRIIA, promoting the phosphorylation and assembly with the low-affinity type I receptor ALK- (activin receptor like-kinase-) 4 or ALK-5 [15, 64].

Myostatin has been reported as a negative regulator of skeletal muscle mass: low levels of myostatin or the knockout of the myostatin gene contributes to muscle mass growth in mice [15–17]. Additionally, the administration of soluble ActRIIB and the overexpression of activin receptor II B (ActRIIB), a dominant-negative form of the myostatin receptor, induce muscle hypertrophy, while increased levels of myostatin lead to skeletal muscular depletion [15, 17, 65].

Some reports have shown that myostatin signalling is enhanced in the skeletal muscle of tumour-bearing rats and mice [13, 14].

In this sense, it has been demonstrated that myostatin is abundantly secreted by C26 colon cancer cells, and it has been verified that the treatment of differentiated C2C12 myotubes with C26-conditioned medium promoted myotubular atrophy and enhanced the activity of the ubiquitin-proteasome pathway. Additionally, the addition of antagonists to myostatin prevented C26-conditioned medium-induced wasting in muscle cell cultures. The authors suggested that myostatin secretion by cachexia-inducing neoplasm would initiate the pathogenesis of cancer cachexia [66].

Likewise, myostatin inhibition by the administration of an ActRIIB/Fragment-crystallizable (ActRIIB/Fc) fusion protein or the ActRIIB soluble form by antisense oligonucleotides is able to prevent the development of muscle mass depletion in tumour-bearing mice [67, 68].

## 5. Leptin

Leptin plays a central role in the control of body weight and energy homeostasis, but it is a pleiotropic cytokine with activities in many peripheral cell types. Several studies in animals and humans have demonstrated the role of leptin in the regulation of energy homeostasis, neuroendocrine function, metabolism, immune function, and bone metabolism. Despite being produced predominantly in adipose tissue, other tissues express leptin, such as placenta, ovaries, mammary epithelium, bone marrow, and lymphoid tissues [69]. Leptin binds to receptors located throughout the central nervous system and peripheral tissues, with at least six receptor isoforms identified (LepRa-f) [70].

In the cancer context, hypoxic conditions often occur in solid tumours. Cellular hypoxia induces hypoxia-induced factor-1 (HIF-1) which activates the leptin gene promoter in human adipocytes and fibroblasts [71].

There is accumulating evidence that leptin signalling might be involved in the development of several types of cancer, such as colon cancer, mammary cancer, prostate cancer, and epithelial ovarian cancer, as well as the development of several myeloid and lymphoid leukemic cell lines. In support of these data, the expression of LR was detected in all these cancer tissues and cell lines [18, 72].

Indeed, leptin has been shown to regulate neoangiogenesis; enhance endothelial cell growth [19, 20]; suppress apoptosis through a Bcl-2-dependent mechanism [73]; act as a mitogen, transforming factor, or migration factor for many different cell types, including smooth muscle cells [74], normal and neoplastic colon cells [75, 76], and normal and malignant mammary epithelial cells [77, 78]; and induce the metastasis of breast cancer, possibly in an autocrine manner [79].

In patients with cancer cachexia, the plasma levels of leptin were lower than in patients without cachexia, which could be due to reduced fat mass in these patients [80]. Because the classic effects of leptin are stimulating *α*-MSH neurons and inhibiting NPY neurons, decreasing food intake, and increasing energy expenditure [56], a leptin-independent pathway of cancer cachexia has been proposed.

Along with low plasma leptin levels, animal models of cancer cachexia may also have an increased number of LRb receptors, which seems to be consistent with the severity of the body fat reduction present in this condition [81]. Moreover, hypoxia-induced factor 1 (HIF-1), which is elevated in cancer cachexia, induces an increase in LRb expression in tumour cells [82].

As stated by Garofalo and Surmacz (2006) [18], it is possible that the local leptin concentration and signalling could be involved in the stimulation of tumour progression. In addition, they suggested that the tumour and surrounding adipose tissue promote a leptin-rich environment, which could contribute to tumour development. Likewise, leptin could contribute to tumour metastasis.

## 6. HIF

As mentioned before, a hypoxic environment is present in different types of tumours, especially in solid tumours. To adjust to the hypoxic microenvironment, several cancer cells increase the production of hypoxia-inducible factors (HIFs). These factors are associated with increased malignancy, poor prognosis, and resistance to radiotherapy and chemotherapy [83]. However, Ranasinghe et al. (2014) [84] reported that prostate cancer cells overexpressed HIF1*α* even under normoxic conditions.

Hypoxia-inducible factors (HIFs) are transcriptional regulators that mediate the cellular response to low oxygen. HIFs consist of an O_2_-sensitive HIF-*α* (HIF-1*α* or HIF-2*α*) and an O_2_-insensitive HIF-1*β* subunit [85]. HIF-1 is the main mediator of hypoxic adaptation, but several tissues and different cell types express both HIF-1 and HIF-2 isoforms under hypoxia [86].

The accumulation of HIF-1*α* promotes the induction of several gene targets, such as leptin and the leptin receptor in tumour cells [87, 88], insulin-like growth factor-binding protein-1, vascular endothelial growth factor A [89], angiopoietin-2, angiopoietin-like 4, plasminogen activator inhibitor-1, glucose transporter-1, hexokinase-2, and glyceraldehyde-3-phosphate dehydrogenase [90]; it also seems to interfere with the transcription of Cdc6 and C-Myc during the regulation of the cell cycle [91].

The literature provides studies that focus on tumour cells that alter the transcriptional profiles via hypoxia-related mechanisms to modulate glycolysis, proliferation, angiogenesis, apoptosis, and metastasis, as to persist under conditions of hypoxic stress [21]. Under hypoxia the induction of glycolysis, angiogenesis, and metastasis seems to be a tumour cell adaptation to survival, which has HIF-1 as a main regulatory factor [27].

The tumour cell in hypoxia also increases the expression of macrophage chemoattractants such as VEGF, endothelins, IL-8, and endothelial monocyte activating polypeptide II (EMAP II) which promoted an increase in monocytes infiltration and macrophages accumulation, especially in tumour avascular or perinecrotic regions [28].

**Figure 1:**
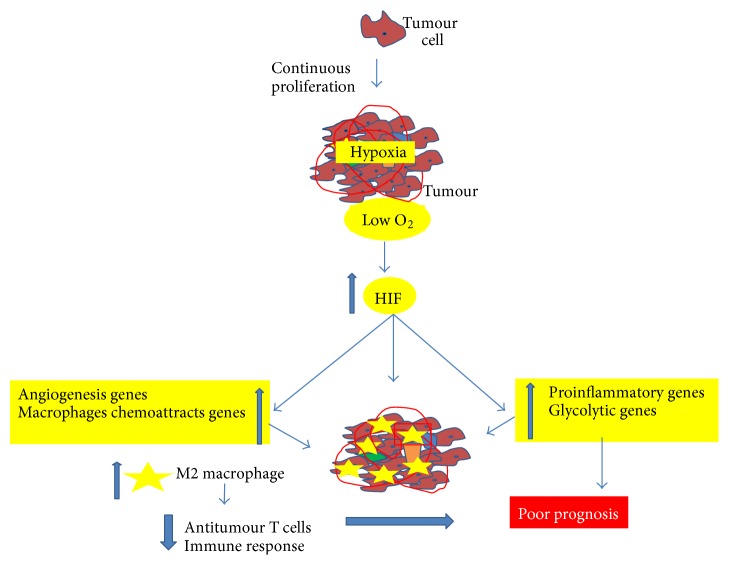
Schematic illustration of the effect of hypoxia on tumour gene expression, macrophage infiltration and antitumour immune response.

In addition, HIF-1 induces myeloid-derived suppressive cells (MDSC) differentiation to tumour-associated macrophages, causes a polarization of M1/M2 type with an increase of M2 in the hypoxia tumour region, and inhibits antitumour T cells, decreasing the immune response [29] (Figure 1).

Using animal models, Liao et al. (2007) [25] demonstrated that the depletion of HIF1-*α* did not impair mammary tumour formation, though decreasing the tumour progression and metastasis. In spite of that, Mazumdar et al. (2010) [22], employing a KRAS-driven lung tumour model, demonstrated that HIF1*α* deletion presents a very small effect on tumour weight and progression, whereas the loss of HIF2*α* actually increased tumour growth and progression.

Studies demonstrated that HIF-1 is implicated in the regulation of several genes involved on multiple key steps of metastasis, including epithelial-mesenchymal transition (EMT), invasion, extravasation, and metastatic niche formation, mostly in solid tumours (for details see review by Liu et al. (2015)) [26].

HIF also contributes for the proinflammatory macroenviroment present in several cancer patients. It is well-known that HIF-1 increases in the adipose tissue of obese individuals inducing the expression of proinflammatory adipokines such as IL-6, leptin, TNF-alpha, and angiopoietin 4, which are involved in the promotion of cachexia and metastasis [23, 24].

## 7. Cancer and Inflammation

The proinflammatory environment can increase the risk of cancer by providing bioactive molecules, including cytokines, growth factors, and chemokines that facilitate carcinogenesis programs and sustain cell proliferative rate, inhibit apoptosis, and stimulate angiogenesis, and extracellular matrix-modifying enzymes, such as metalloproteinases, which promote the epithelial-mesenchymal transition (EMT).

## 8. IL-6

There is evidence that IL-6 is implicated in promoting tumour growth metastasis and participates in the development of cancer cachexia. IL-6 is considered the prime regulator of the acute-phase response in cachectic patients.

In the 1990s, there was increasing evidence that IL-6 contributes to metastasis and that serum IL-6 levels are adverse prognostic factors for the development of metastasis in several tumour types [30, 31].

Chang et al. (2013) [32], using* in vivo* and* in vitro* experiments, analysed the effects of IL-6 on cancer development and demonstrated that there is a positive correlation between IL-6 and human mammary tumour development and metastasis, which seems dependent on STAT-3. The authors proposed the “formation of an autocrine/paracrine IL-6/JAK/STAT3 feed-forward loop, which participates in tumour proliferation, shaping of the tumour microenvironment, and metastasis.”

Recently, Guyer and Macara (2015) [92] showed that IL-6 is important for inducing STAT3 in mammary epithelial cells downstream of silencing the cell polarity protein Par3, an important regulator of mammary tissue structure, which protects the development of primary tumour growth and aggressive metastatic lesions.

IL-6 signalling involves the binding of the cytokine to the membrane-bound IL-6 receptor (mIL-6r) on target tissues, which include hepatocytes, immune cells, and skeletal muscle. The activation of mIL-6r consequently promotes the downstream activation of many signalling pathways, including JAK/STAT3 and p38. Several of these pathways have also been implicated in the regulation of muscle mass loss during cancer cachexia [93].

Batista et al. (2013) [94] evaluated the correlation between adipokine tissue expression and concentrations in cachectic and noncachectic patients with or without cancer. They found that the plasma concentration of IL-6 was 11.4 times higher in the cachectic cancer group compared with the groups without cancer and with healthy weight, in addition to demonstrating a significant correlation with the presence of cancer. The interaction between cachexia and tumours increased the amount of IL-6 in subcutaneous adipose tissue and increased the IL6/IL-10 ratio, but not in visceral adipose tissue. Thus, they suggested that subcutaneous adipose tissue is associated with changes in plasma adipokines, which can play a role as markers of cachexia. However, a more specific study of the adequacy of the IL6/IL-10 ratio in the setting of cancer cachexia is necessary.

Suh et al. (2013) [95] evaluated 98 advanced cancer patients and observed that IL-6 may be a good indicator of survival time in patients with advanced cancer in later life, despite elevated IL-6 previously being considered an indicator of shorter survival in these patients. Additionally, Kim et al. (2012) [96] investigated the roles of proinflammatory cytokines in lung cancer and colorectal cancer patients with cachexia prior to treatment. They observed that patients with high levels of IL-6 showed >5% weight loss after 6 months, suggesting that IL-6 could be responsible for the induction and maintenance of cancer cachexia.

Puppa et al. (2011) [97] demonstrated in tumour-bearing mice an association among increases in gut permeability, endotoxemia, and plasma IL-6 concentration with tumour growth and cachexia development.

Bonetto et al. (2012) [33] suggested that STAT3 is a primary mediator of muscle mass loss because STAT3 activation in skeletal muscle by elevated IL-6 family ligands appears to be necessary and sufficient to promote muscle mass loss, in addition to being a common characteristic observed* in vivo* and* in vitro* and for different types of cancer. However, it is not yet clear how the activation of STAT3 promotes muscle atrophy. Haddad et al. (2005) [34] showed that the infusion of IL-6 in muscle reduces the phosphorylation of S6K1, which is associated with a catabolic process. S6K1 is phosphorylated and activated by mTOR, and a reduction in the phosphorylation of S6K1 is associated with the loss of the cellular capacity to synthesize proteins. Additionally, as described above, IL-6 seems to be involved in decreasing body fat and food intake by acting on both energy expenditure and NPY release control in the hypothalamus arcuate nucleus [35].

From these reports the idea that IL-6 could be a good marker to predict the evolution of cachexia associated with cancer and could be employed in treatment strategies emerged. Moreover, it was suggested that treatments that could impair the increase in IL-6 in adipose tissue could ameliorate cachexia in cancer. More studies are essential to elucidate this issue.

In this sense, it has been demonstrated that endurance exercise ameliorates cachexia-related inflammation in a rodent model, causing a systemic effect that is also associated with adipose tissue and decreases IL-6 in mesenteric adipose tissue [98, 99]. Likewise, other studies using anti-IL-6 antibodies, either* in vitro* or* in vivo*, showed improvements in cancer cachexia [100, 101].

It is important to note that although IL-6 has an important effect on the development of cachexia associated with cancer, IL-6 cannot be considered the only factor contributing to the breakdown of skeletal muscle and, consequently, to the development of cachexia [38, 102].

Some cancer patients present high serum levels of TNF-a, IL-6, and IL-1 which correlate positively with the progression of some tumours. It has been postulated that these cytokines promote anorexia in cancer by enhancing the levels of corticotrophin-releasing hormone, a central nervous anorexigenic neurotransmitter, causing a decrease in food intake [36].

Schéle et al. (2013) [35], using IL-6^−/−^ and IL-1R1^−/−^ mice, suggested that both endogenous IL-1 and IL-6 could suppress the expression of NPY and agouti-related protein in the hypothalamic arcuate nucleus. As stated by Trayhurn and Bing (2006) [103], IL-6 is the most interesting adipokine evolved in the regulation of energy balance. IL-6 induces weight loss [104], and it is expressed with its receptor in the neurons of the hypothalamic nuclei that regulate energy homeostasis [105]. In this sense, Wallenius et al. (2002) [37] demonstrated that the chronic ICV administration of IL-6 reduces body fat through an upregulation of energy expenditure without causing an acute-phase reaction.

## 9. TNF-*α*

Tumour necrosis factor (TNF-*α*) is an inflammatory mediator present in the tumour microenvironment that has been implicated in carcinogenesis, especially in the early stages, including angiogenesis and invasion, versus the progression of carcinogenesis [40]. TNF-*α* is the main inflammatory cytokine that induces a transcription factor, Snail, that is implicated in EMT induction and stabilization [106].

Furthermore, studies have proposed that systemic TNF-*α* might also be involved in the early development of some tumours. As reported by Balkwill (2006) [107], several studies using TNF-*α*- and TNF-R1-knockout mice and a variety of cell cultures have demonstrated the role of TNF-*α* in cancer development.

Recently, studies showed elevated TNF-*α* plasma levels to be associated with an increased risk of colorectal adenomas and the development and progression of breast tumours [108, 109].

However, the results regarding the role of TNF-*α* in cancer are controversial; high concentrations of this cytokine induced an antitumoural response in a murine model of sarcoma [110]. In contrast, low, sustained TNF-*α* production levels can induce a tumour phenotype [107].

Obesity is linked with chronic, subclinical inflammation characterized by elevated levels of circulating proinflammatory mediators produced by adipose tissue, such as leptin, TNF-alpha, and IL-6 [111]. This inflammatory condition could be involved in an increased risk of cancer development. In fact, several authors have reported a correlation between obesity and cancer development (see review Calle and Kaaks (2004)) [2].

Furthermore, TNF-*α* seems to be involved in the progression of cancer cachexia. As previously noted, cachexia is related, among other factors, to decreases in fat mass and skeletal muscle associated with negative protein synthesis and positive proteolysis. Muscle atrophy is characterized by a reduced cross-sectional area of myofibers accompanied by a loss of strength and a change in the composition of muscle fibre types. During the process of cancer cachexia, rapid type II fibres are affected, contributing to a higher proportion of slow fibres compared to fast fibres. In this case, catabolic factors, such as IL-6 and TNF-*α*, are increased, contributing to the loss of muscle mass, and it has been suggested that the tumour has a great influence on the increase in the circulation of these factors [38, 39]. In fact, it has been demonstrated that TNF-*α* acts more on type II fibres to stimulate apoptosis signalling [41].

Several factors, including TNF-*α* and IL-6, are associated with protein degradation by ubiquitination and the proteasome pathway, the most predominant pathway among the pathways of protein degradation. TNF-*α* is able to increase the expression of ubiquitin and to promote the accumulation of ubiquitinated proteins, contributing to the atrophy of muscle mass [112, 113]. The activation of NF-kB is a major candidate as the mediator of the cellular response of TNF-*α* after TNF-*α* stimulates the activation and nuclear translocation of NF-kB in skeletal muscle cells, which contributes to muscle catabolism [38]. The p38/MAP kinase pathway can also be stimulated by TNF-*α*. Both p38/MAP kinase and NF-kB upregulate the expression of genes that encode the E3 ligases MuRF1 and MAFbx, thus inhibiting protein synthesis and contributing to muscle atrophy [114].

Although the role of TNF-*α* during the inflammatory process in cachexia induced by cancer has been extensively studied, there is no consensus about the degree of influence of this cytokine on this inflammatory process [115]. It appears that the levels of serum TNF-*α* are related to cancer stage, which reflects the size of the tumour [116]. Corroborating the result mentioned above, Kemik et al. (2012) [117] conducted a study with the aim of evaluating acute-phase proteins, cytokines, and hormones in cachectic patients with various cancers of the gastrointestinal tract. They found higher serum concentrations of several factors, including TNF-*α* and IL-6, in patients with oesophageal, gastric, pancreatic, colon, and rectal cancers than in controls. Thus, the authors suggested an association between proinflammatory cytokines in cachectic patients with various types of gastrointestinal cancer. However, other authors have found different results in patients with other types of cancer. Gulen et al. (2012) [118] studied 63 patients with lung cancer cachexia with advanced disease with the aim of evaluating the relationship between adipokines and systemic inflammation in patients at this stage. The authors showed the absence of a relationship between adipokines or systemic inflammation and cancer cachexia in lung cancer patients: some proinflammatory factors, such as TNF-*α*, did not differ from the control group.

Some authors have shown that TNF-*α* correlates with body mass, but others have shown no correlation between this cytokine and anorexia in patients with advanced-stage cancer [102].

Amaral et al. (2006) [42] demonstrated in rat that the administration of TNF-*α* in the hypothalamus promoted a decrease in 12-hour food intake by modulating the expression of neurotransmitters associated with energy balance, favoriung higher energy expenditure. Previously, Aguilera et al. (1998) [119] observed a negative correlation between NPY and TNF-*α* in patients with nervous anorexia.

According to Arruda et al. (2010) [120], a high concentration of TNF-*α* also acts in the expression of hypothalamus-modulating neurotransmitters and signal transduction pathways, thus increasing body temperature, oxygen consumption/carbon dioxide production, and energy expenditure and contributing to weight loss in cancer cachexia patients.

Busquets et al. (1998) [121] demonstrated in rat that the administration of a single intravenous injection of TNF-*α* increased the gene expression of uncoupling proteins 2 and 3 in skeletal muscle, suggesting this as a possible mechanism contributing to increased energy expenditure and cachexia in tumour-bearing cancers. However, weight loss in pancreatic cancer is associated with systemic inflammation and increased ubiquitin mRNA expression, but not uncoupling proteins in skeletal muscle [122].

An* in vitro* study showed that, in 3T3-L1 adipocytes, TNF-*α* significantly reduced the lipid accumulation and glucose uptake induced by adiponectin, and it increased lipolysis [123]. Previously, it was demonstrated in humans that the administration of TNF-alpha at low doses caused systemic lipolysis [124]. This finding also suggests a potential role of this cytokine in the control of adipose tissue depletion in cancer cachexia.

Another factor recently described to be involved in metabolic regulation is ANGPT4. This protein is associated with energy homoeostasis, wound repair, tumourigenesis, angiogenesis, and redox regulation [43].

## 10. Angiopoietin-Like 4 (ANGPTL4)

A new protein with similarity to members of the ANG (angiopoietin) family was identified by three independent research groups in 2000 [125, 126]. ANGPTL-4 was also named fasting-induced adipose factor (FIAF) [127].

The protein ANGPTL4 was classified as an adipokine because it was expressed predominantly in adipose tissues and liver; thus, it was believed to be involved in lipid metabolism [43, 128]. ANGPTL4 primarily showed an inhibitory effect on LPL activity [125, 129].

ANGPTL4 expression is also found in skin, intestines, kidneys, adipose tissues, liver, and a variety of tumours [125, 126, 130]. ANGPTL-4 is cleaved by proprotein convertases, releasing nANGPTL4 and the C-terminal portion of ANGPTL4 (cANGPTL4) [131, 132].

An analysis of recent studies makes it clear that the specific form of ANGPTL4 associated with the microenvironment tumour influences the clinical impact of this protein, along with other factors [44, 130, 133].

Over the past decade, there has been increasing recognition that this protein participates in numerous physiological and pathological processes [130]. ANGPTL-4 has been implicated as an important factor involved in energy homoeostasis, redox regulation, angiogenesis, the development of cancer and cachexia, and the development of metastasis and inflammation; however, the studies are still incomplete and often contradictory [43].

Galaup et al. (2006) [134] found in an experimental model that Angptl4 prevents tumour metastasis through the inhibition of vascular permeability, tumour cell motility, and invasiveness. Similarly, Yang et al. (2008) [131] demonstrated that cANGPTL4 inhibits angiogenesis. Because angiogenesis plays an important role in the progression of cancer, the authors suggested that cANGPTL4 has an antitumoural effect, thus decreasing tumour growth and metastasis.

In contrast, hypoxia stimulates the expression of ANGPTL4 in several tumour types [43, 135]. ANGPTL4 mRNA is increased in the perinecrotic areas of many human tumours [135, 136]. Moreover, elevated ANGPTL4 expression increased as tumours progressed from benign to metastatic states, thus implying a role of ANGPTL4 in tumour growth [45, 46].

The Angptl4 protein is more often expressed in colorectal cancer (CRC) tissues than that in normal tissues, and it is related to cell migration through the cytoskeletal signalling pathway [44]. The overexpression of Angptl4 promotes colon cancer cell migration through the cytoskeletal signalling pathway, while the downregulation of ANGPTL4 impairs tumour growth and metastasis [44, 46, 47].

Hypoxia, fasting, and PPARs regulate ANGPTL-4 expression [46, 137].

Inflammatory factors, such as IL-1b and IL-6 or hypoxia alone, increased ANGPTL4 protein levels, but the role of hypoxia was more significant. It was also observed that increasing tumour size and increasing degree of hypoxia in the tumour mass promoted the upregulation of ANGPTL4, especially in the hypoxic areas surrounding the necrotic area [138].

Studies have shown that ANGPTL4 is a target gene of PPARs, which are involved in the development of tumours [139, 140]. However, the role of PPARs in the regulation of ANGPTL-4 in tumour cells is controversial. Zhu et al. [46] (2011) did not detect a correlation between PPAR *α*, *γ*, and *β* and the expression of ANGPTL-4. In contrast, Girroir et al. (2008) [137] demonstrated that the addition of PPAR *γ* and *β* to tumour cell culture (MCF7 and UACC903) reduced cell proliferation and increased ANGPTL-4 gene expression.

The metabolic effects of ANGPTL-4 support the idea that this protein contributes to cancer cachexia development: fat-specific Angptl4 overexpression caused a 50% reduction in adipose tissue weight, partly by enhancing fatty acid oxidation and lipolysis [48]; the central administration of ANGPTL4 lowered food intake and body weight gain and enhanced energy expenditure [49].

## 11. Conclusion

The grade of malignancy of tumour is primordially associated with both the development of metastatic lesions and cancer-associated cachexia, which are multifactorial conditions depending on factors present in the micro- and macroenvironments in the tumour-bearing patient.

**Figure 2:**
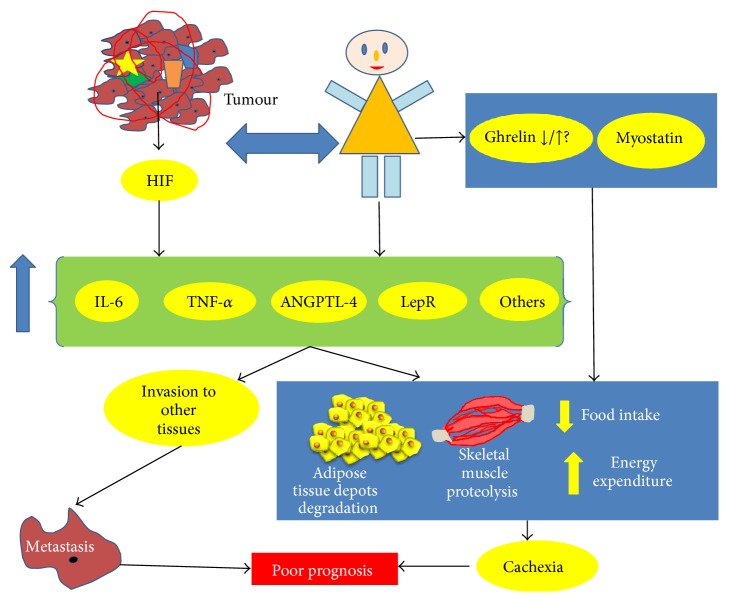
Schematic illustration of the cross talk between micro- and macroenvironment in the tumour-bearing patient. The importance of hypoxia and proinflammatory factors for tumour metastasis and cancer cachexia.

In this review, we point out some factors that are released and/or act in the tumour and other tissues related to the development of metastasis and cancer cachexia. Hypoxia-induced factor plays a prominent role in inducing tumour macro- and microenvironment release of proinflammatory cytokines. The cross talk among factors released by the tumour, adipose tissue, muscle, and other tissues seems to contribute to the difficulty of treating the cancer patient, once a multifactorial alteration occurs in the micro- and macroenvironment of the individual (Figure 2) (Table 1). Furthermore, there is still some controversy in the literature, related to the role of ghrelin, ANGPT4, HIF-1, and TNF-*α* in cancer cachexia and metastasis development, indicating the necessity of more studies.

## Figures and Tables

**Table 1:** Summary of the effect of some factors involved in the development of cancer cachexia and metastasis.

Factor	Action	Reference
Ghrelin	↑ in cachexia cancer patients	[11]
	↓ Myostatin	[12]

Myostatin	↑ in Cancer/Cachexia	[13, 14]
	↑ Proteolysis	[15–17]

Leptin	Contribute to metastasis	[18]
	Regulate neoangiogenesis	[19, 20]

HIF	↑ Apoptosis	[21]
	↑ Angiogenesis	[21]
	↑ Proliferation tumour cells	[22]
	↑ IL-6, leptin, TNF-*α*, and ANGPTL-4	[23, 24]
	Contribute to metastasis	[23–26]
	↑ Glycolysis	[27]
	↑ VEGF, endothelins, IL-8, and EMAP II	[28]
	↑ M2 ↓ T cells response	[29]

IL-6	Contribute to metastasis	[30–32]
	↑ Proteolysis and atrophy muscle mass	[33, 34]
	↓ Food intake and ↑ energy expenditure	[35–37]

TNF-*α*	↑ Proteolysis	[38, 39]
	Contribute to angiogenesis	[40]
	Stimulate apoptosis	[41]
	↓ Food intake and ↑ energy expenditure	[42]

ANGPTL-4	Contribute to angiogenesis	[43]
	Contribute to metastasis	[43–47]
	↑ Lipolysis and fatty acid oxidation	[48]
	↓ Food intake and body weight gain	[49]
